# *Porphyromonas gingivalis* outer membrane vesicles exacerbate retinal microvascular endothelial cell dysfunction in diabetic retinopathy

**DOI:** 10.3389/fmicb.2023.1167160

**Published:** 2023-05-11

**Authors:** Shengyuan Huang, Guoqin Cao, Dong Dai, Qiuping Xu, Sunniva Ruiz, Satoru Shindo, Shin Nakamura, Toshihisa Kawai, Jiang Lin, Xiaozhe Han

**Affiliations:** ^1^Department of Stomatology, Beijing Tongren Hospital, Capital Medical University, Beijing, China; ^2^Department of Oral Science and Translation Research, College of Dental Medicine, Nova Southeastern University, Fort Lauderdale, FL, United States

**Keywords:** diabetic retinopathy, endothelial dysfunction, *Porphyromonas gingivalis*, outer membrane vesicles, protease-activated receptor 2

## Abstract

Diabetic retinopathy (DR) is one of the leading causes of blindness. Periodontitis is one of the highest oral incidences and has been closely related to various systemic conditions through *Porphyromonas gingivalis* (*P. gingivalis*). *P. gingivalis* OMVs, derived from *P. gingivalis*, can cause endothelial dysfunction and potentially affect microvascular diseases. Current epidemiological studies provide limited evidence suggesting that periodontitis is associated with DR. However, there is a lack of basic research elucidating how periodontitis affects the severity of DR. This study aimed to explore the potential of *P. gingivalis* OMVs to contribute to the pathogenesis of DR and explore how it affect the retinal microvascular endothelium. The results demonstrated that *P. gingivalis* OMVs accelerated the blood-retinal barrier damage in DR mice. *In vitro* studies showed that the expression of inflammatory factors in human retinal microvascular endothelial cells (HRMECs) was increased after *P. gingivalis* OMVs stimulation, and the increased reactive oxygen species production, mitochondrial dysfunction, apoptosis, and altered endothelial permeability were observed in HRMECs under *P. gingivalis* OMVs stimulation. In addition, we found that protease-activated receptor-2 (PAR-2) regulated OMVs-induced TNF-α, MMP-9 mRNA expression, cell death, and endothelial permeability. Overall, we suggested that *P. gingivalis* OMVs induced mitochondria-related cell death of HRMECs and accelerated endothelial dysfunction, thus aggravating DR, in which PAR-2 plays a potential role. This study is the first research report to delineate the potential molecular mechanism of *P. gingivalis* OMVs on DR pathogenesis, which uniquely focused on elucidating the possible impact of periodontal pathogen derivatives on DR progression.

## 1. Introduction

Diabetic retinopathy (DR) is one of the most common microvascular complications of diabetes mellitus (DM). Because of its irreversible damage to visual function, it is one of the leading causes of blindness in humans. In all types of diabetic patients, the prevalence of DR is 22.27% (Yau et al., 2012; Teo et al., 2021). The rapidly growing global aging population, the increasing lifespan of people living with DM, and diet habit changes lead to an increased risk for DR. It is predicted that the global DR population will increase by 55.6% from 2020 to 2045, reaching 700 million (Teo et al., 2021). While the classic risk factors for DR are hyperglycemia, hypertension, dyslipidemia, etc., there is evidence that many DM patients without these risk factors still develop DR. Study has shown that glycosylated hemoglobin accounts for only 6.6% of the risk of DR (Hirsch and Brownlee, 2010). Therefore, identification of new DR risk factors remains critical for disease prevention and treatment. The fundamental cause of DR pathogenesis is the disorder of microcirculation. Among a variety of complex factors, oxidative stress-mitochondrial damage and increased inflammation are considered to play a critical role in the development of this blinding disease (Forrester et al., 2020). Increased levels of oxidative stress lead to intracellular production of high levels of reactive oxygen species (ROS), leading to cellular inflammation, mitochondrial damage, and release of proapoptotic substances (Kang and Yang, 2020). On the other hand, pro-inflammatory cytokines, such as TNF-α, IL-1β, IL-6, IL-8, and ICAM-1, play a promoting role in inflammatory response in DR (Forrester et al., 2020).

Periodontitis is one of the chronic diseases with the highest oral incidence and has been closely related to various systemic conditions (Nazir, 2017). Current clinical and epidemiological studies provide preliminary evidence suggesting that periodontitis is associated with diabetic retinopathy (Rosenthal et al., 1988; Noma et al., 2004; Amiri et al., 2014; HR et al., 2018). However, there is a lack of mechanistic research in this area elucidating how periodontitis affects the severity of DR. *Porphyromonas gingivalis* (*P. gingivalis*) is one of the key pathogens in periodontitis and has been suggested playing a crucial role in vascular diseases (Deshpande et al., 1998). With the advancement of the related research on this microorganism, *P. gingivalis*-derived Outer Membrane Vesicles (*P. gingivalis* OMVs) has gained increased attention on its potential pathogenicity. Furthermore, OMVs is stable because they have an intact membrane structure and are not affected by host-derived proteases (Zhang et al., 2020). Studies have shown that *P. gingivalis* OMVs can cause endothelial dysfunction and is associated with cardiovascular disease (Farrugia et al., 2020). The pathogenesis of DR is characterized by changes in the endothelial dysfunction of the retinal microvascular endothelium with increased levels of adhesion molecules and inflammatory factors (Yang et al., 2022). These series of changes demonstrate similarities with other vascular diseases affected by *P. gingivalis* OMVs. Therefore, *P. gingivalis* OMVs may contribute to DR pathogenesis through inducing endothelial dysfunction.

Protease-Activated Receptor 2 (PAR-2) is a cell surface protein that is widely expressed in various tissues, including retinal and gingival tissues (Kumar VR et al., 2016; Rovai and Holzhausen, 2017). PAR-2 is known to be involved in various physiological processes including inflammation, angiogenesis, and wound healing. Studies have shown that PAR-2 may be involved in the pathogenesis of DR. Activation of PAR-2 leads to increased inflammation, which is a key feature of diabetic retinopathy (Zhu et al., 2006). Interestingly, *P. gingivalis* can activate PAR-2, leading to increased periodontal inflammation and bone resorption (Holzhausen et al., 2006). *P. gingivalis* OMVs are derived from *P. gingivalis* and contain most of the virulence factors of *P. gingivalis*. However, whether *P. gingivalis* OMVs can activate PAR-2 and accelerate the development of DR pathogenesis remains unknown.

The purpose of this study was to elucidate the molecular mechanism of *P. gingivalis* OMV-induced retinal microvascular endothelial cell dysfunction and investigate the effect of *P. gingivalis* OMVs on the pathological development of DR in a mouse model. Our results demonstrated that *P. gingivalis* OMVs exacerbate microvascular dysfunction *in vivo* and induce human retinal endothelial cell death *in vitro*. This study provided new insight into the potential link between periodontitis and pathological process of DR, and paved way for future comprehensive studies of the impact of periodontal pathogen-derived OMVs on DR and other systemic diseases.

## 2. Materials and methods

### 2.1. *Porphyromonas gingivalis* bacteria culture and OMVs isolation and quantification

*Porphyromonas gingivalis* 33277 (ATCC, United States) was subcultured in tryptic soy broth (TSB) medium with an anaerobic environment at 37°C for 24 h. The protocol of *P. gingivalis* OMVs isolation was modified according to the previously described procedure (Farrugia et al., 2020). Cultured bacteria were filtered through 0.22 μm polyvinylidene fluoride (PVDF) filters (Millipore, United States), then centrifuged at 160000 × g for 3 h at 4°C. The pellet was washed once with PBS and centrifuged at 160000 × g for 3 h at 4°C. According to the manufacturer’s instructions, BCA Protein Assay Kit (Thermo Scientific, United States) was used to quantify the protein concentration. The sizes of *P. gingivalis* OMVs particles were measured by NanoSight NS300 (Malvern Panalytical, U.K).

### 2.2. Animal model and treatment

Six-week-old male C57BL6/J mice (Vital River Laboratory, China) were housed in the SPF-level laboratory and were randomly divided into four groups: Control, Streptozotocin (STZ), OMVs, and STZ + OMVs (*n* = 8). The mice diabetes model was induced by intraperitoneal injection of 120 mg/Kg STZ. Three days after the injection, the blood glucose was determined by tail pricking with a blood glucose meter for three consecutive days (Piano et al., 2016). The mice with blood glucose lower than 16.7 mol/L were excluded. After 12 weeks, *P. gingivalis* OMVs (100 μg) was injected through the tail vein twice a week for 8 weeks according to previous study (Gong et al., 2022). This animal study was approved by the Institutional Animal Care and Use Committee of Capital Medical University (Beijing, China).

### 2.3. Optical coherence tomography (OCT)

OCT was performed on the Micron III OCT module (Phoenix Research Laboratories, United States). Mice were anesthetized by intraperitoneal injection of ketamine (80 mg/kg) and xylazine (10 mg/kg), then dilated with 1% tropicamide eye drops and covered with ofloxacin eye ointment. The retina was scanned along the same vertical axis.

### 2.4. Fundus fluorescein angiography (FFA)

Pathological changes in the fundus were detected using a Micron III retinal imaging microscope (Phoenix Research Laboratories, United States). The mice were stabilized on the microscope platform, and images were recorded after intraperitoneal injection of fluorescein sodium (10%, 75 mg/kg).

### 2.5. Retinal digestion

Mice were euthanized, eyeballs were collected and fixed in 4% paraformaldehyde. The retina was separated and digested by 1 mg/ml proteinase K and 3% trypsin for 2 h. Retinal microvascular tissue was transferred to glass slides. Entirely dried the slides and stained with hematoxylin/eosin solution. Images were taken using a microscope. For each retinal slide, pericyte and acellular capillary counts were performed in randomly selected regions of interest according to the evaluation criteria shown in Supplementary Figure S1. ImageJ software was used to measure microvessel diameter. Briefly, the regions of interest were selected from retinal slides randomly. The “Angle tool” was used to draw a line L1 along the direction of the microvessel, the mouse was moved along the vertical direction of L1 and stopped at the opposite edge of the microvessel to get a straight line L2. The angle between L1 and L2 was measured and the direction of L2 was adjusted to make the angle between L1 and L2 as close to 90 degrees as possible. The angle between L1 and L2 was saved. The length of L2 was defined as microvessel diameter and measured for 3 times. Mean microvessel diameter were calculated and finally normalized to micrometer units.

### 2.6. *Porphyromonas gingivalis* arginine gingipain (Rgp) detection by ELISA

The blood and the eyeballs were collected immediately after the mice were euthanized. Isolated the serum. The cornea was pierced with a syringe to aspirate the aqueous humor. Retinal tissue was thoroughly homogenized, followed by centrifugation and supernatant collection. Rgp concentration was detected by Rgp specific ELISA kit (PYRAM, China) according to the manufacturer’s instructions. The ELISA was performed with the SpectraMaxiD3 microplate reader (Molecular Devices, United States) and absorbance values were recorded at 450 nm wavelength.

### 2.7. Cell culture and stimulation

Primary human retinal microvascular endothelial cells (HRMECs) were purchased from Cell systems company (United States) and cultured in MCDB 131 Medium (Thermo Scientific, United States) including 10 ng/mL Epidermal Growth Factor (EGF, Thermo Scientific, United States), 1 μg/mL Hydrocortisone (Sigma-Aldrich, Germany), 10 mM Glutamine (Thermo Scientific, United States), 100 U/mL of penicillin (Gibco, United States), 100 μg/mL of streptomycin (Gibco, United States), 0.25 μg/mL of Amphotericin B (Gibco, United States) and 10% FBS (Gibco, United States). HRMECs were cultured in a 5% CO_2_ incubator at 37°C.

For *P. gingivalis* OMVs stimulation, 0.5 × 10^6^ cells were subcultured in a 6-well plate to 70% confluence. Based on previous studies, we adjusted the *P. gingivalis* OMVs as a final concentration of 50 μg/ml to stimulate HRMECs and incubated for 24 h (He et al., 2020). In some experiment, AZ3451 (Sigma-Aldrich, United States), as a PAR-2 antagonist, was added into the culture at the final concentration of 20 μM. Cells were harvested for testing after 24 h stimulation.

### 2.8. Total ROS assay

For the total ROS assay, Rosup (50 μg/mL) in a separate well was added as the positive control. Fluorescent dye 2′,7′-dichlorodihydrofluorescein diacetate (H2DCFH-DA) was used to analyze the ROS production level in HRMECs. Observe and record under a fluorescent microscope after 30 min of incubation using a total ROS kit (Biosharp, China) and according to the manufacturer’s instructions. Fluorescence intensity analysis was performed with ImageJ.

### 2.9. Mitochondrial membrane potential detection

The mitochondrial membrane potential of HRMECs was measured using a JC-1 kit (Biosharp, China). Briefly, JC-1 dye was added in each well and incubated in a 37°C 5% CO_2_ incubator for 20 min. Cells incubated with 10 μM Carbonyl cyanide 3-chlorophenylhydrazone (CCCP) for 20 min were used as a positive control. The images were recorded using a fluorescence microscope at 530 nm and 595 nm wavelengths.

### 2.10. Dextran leakage assay

The fluorescence intensity of the leaked dextran was analyzed in a Transwell system (Corning, United States) to assess changes in endothelial permeability. The method was modified from previous reports (Ye et al., 2022). Briefly, HRMECs were cultured in the Transwell inserts. 50 μg/mL *P. gingivalis* OMVs were added in the presence or absence of 20 μM AZ3451 in the upper compartment. After 24 h, the culture medium was aspirated and replaced with fresh culture medium containing 100 μg/mL Alexa Flour 488-dextran 3,000 MW (Thermo Scientific, United States) into the upper compartment. After 3 h of incubation, the medium from the lower compartment was obtained and measured by the FilterMax F5 microplate reader (Molecular Devices, United States).

### 2.11. Cell apoptosis by flow cytometry

The ratio of apoptotic cells to total cells was assessed using the violet ratiometric membrane asymmetry probe/dead cell apoptosis kit (Thermo Scientific, United States) followed by flow cytometry. HRMECs were incubated with 4’-N,N-diethylamino-6-(N,N,N-dodecyl-methylamino-sulfopropyl)-methyl-3-hydroxyflavone (F2N12S) and SYTOX AADvanced for 5 min. The data was collected on BD LSRFortessa X-20 (BD Biosciences, United States) according to the manufactory’s recommended settings. The ratio of apoptotic cells was analyzed by Flowjo 10.8.1.

### 2.12. Real-time qPCR

Using TRIzol and PureLink RNA Mini Kit (Invitrogen, United States) for total RNA isolation. RNA was reverse transcribed to complementary DNA (cDNA) using a Verso cDNA Synthesis Kit (Thermo Scientific, United States). SYBR green was used for PCR reaction. The levels of gene expression were calculated using the 2 − ΔΔCt method and normalized to GADPH levels. Sequences of primers used in RT-qPCR are presented in Table.1.

**Table 1:** Primers for RT-qPCR.

Gene	Forward Primer	Reverse Primer
GAPDH	GAAGGTGAAGGTCGGAGTC	GAAGATGGTGATGGGATTTC
ICAM-1	ATGCCCAGACATCTGTGTCC	GGGGTCTCTATGCCCAACAA
Ve-cadherin	TTGGAACCAGATGCACATTGAT	TCTTGCGACTCACGCTTGAC
Caspase-1	AAGCATTCAGGGAAGTAGAGTGAAAATAG	ATCCCTCAGCTGGAGTATAAATACTAGG
Caspase-3	TCACCATGGCTCAGAAGCAC	CAACTTCCTAAAATGGTTTGAGATGTGTT
Caspase-7	TTCCCAACTGCATAGAATGATGTCTG	CTGTGTTTTGATGATGGAATGGACTG
Bcl-2	CCGGGAAGCAACAACTCTGAT	GGATCCAGGATAACGGAGGCT
Bax	TGTTGGGCTGGATCCAAGAC	GGAGGTCAGCAGGGTAGATGA
FASL	CTTGCCTCCTCTTGAGCAGT	CTCCTTTGACCAGGCCTTCTG
FAS	GCACAGCAGATACTGCCAATTT	AATCCCTTGGAGTTGATGTCAGTC
TNF-α	CTCTCCCCTGGAAAGGACAC	GTGGGAGAGTGGATGAAGGC
IL-1β	AGAAGGTGGTTGTCTGGGAATAAG	CACTGCTGTGTCCCTAACCA
MMP-2	AACTACAACTTCTTCCCTCGCAA	AGCCTTCACAAAAGACACCTCAT
MMP-9	GCTTCTCCAGAAGCAACTGTC	ACAGGACATGTTCACCGCT
IL-6	CCTCTAGTGGTGTTTGTTTTAGGGA	ATATGTATACAGGCACTGCATGCAA
TGF-β	GGCCAGATCCTGTCCAAGC	GTGGGTTTCCACCATTAGCAC

### 2.13. Statistical analysis

Data in this study were presented as mean ± standard deviation and analyzed by GraphPad Prism 9. ANOVA was used to analyze differences between groups. An unpaired-***t***-test was used to evaluate the difference between two conditions. Results were considered statistically significant when *p* < 0.05.

## 3. Results

### 3.1. *Porphyromonas gingivalis* OMVs exacerbated DR microvascular pathology *in vivo*

**Figure 1:**
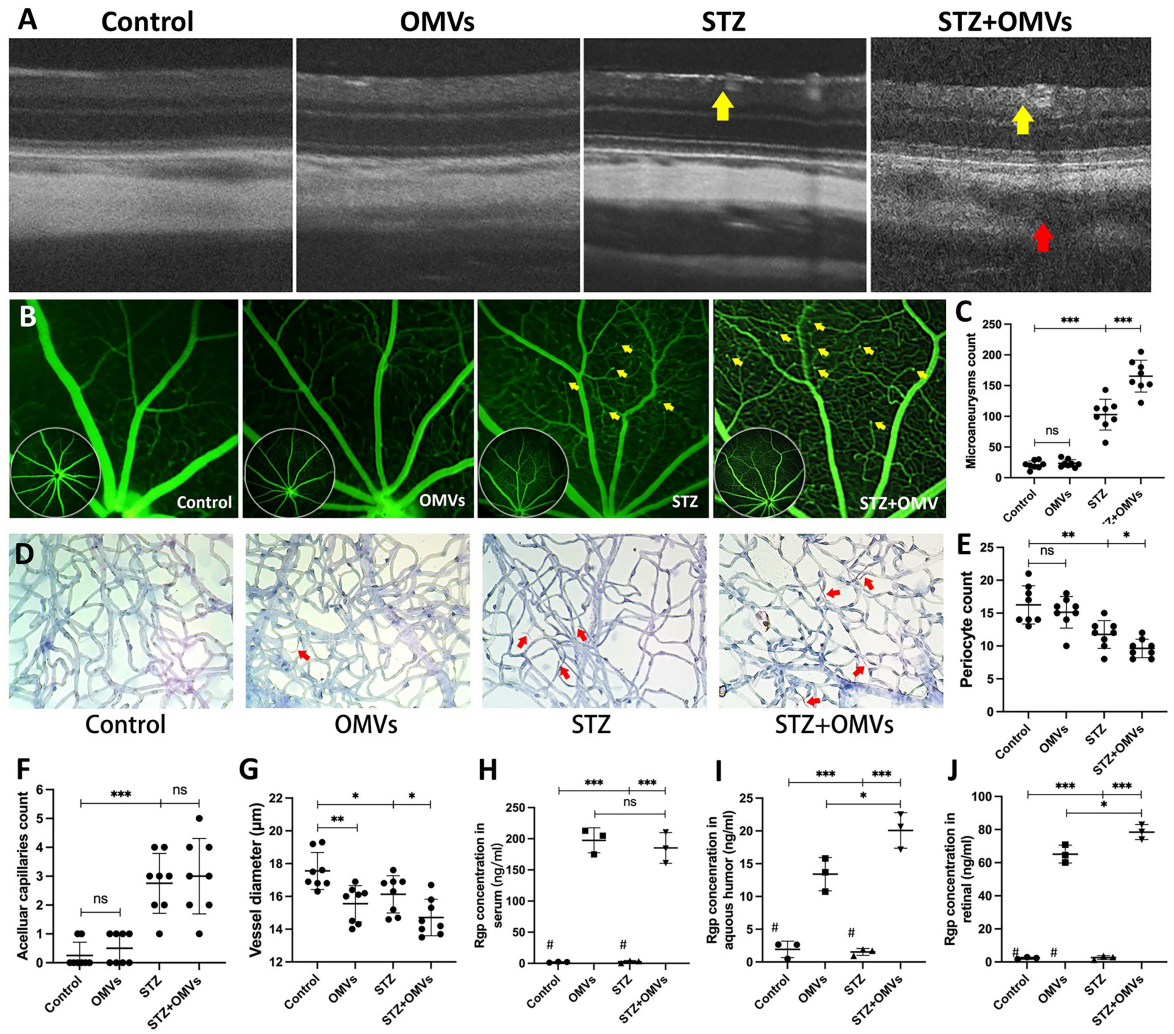
**(A)** The representative fundus OCT images of mice in different groups, hard exudation (yellow arrow); retinal pigment epithelial fluid (red arrow). **(B)** The representative FFA images of mice after intraperitoneal injection of 75 mg/kg 10% fluorescein sodium. The yellow arrows indicate microaneurysms. **(C)** The number of microaneurysms in different groups was calculated based on FFA (*n* = 8). **(D)** The representative images of mice retinal digested slices in different conditions (H/E staining, 200×). **(E–F)** The number of pericytes **(E)**, number of acellular capillaries (red arrow) **(F)**, and Vessel diameter **(G)** in different conditions were calculated by retinal digested slices (*n* = 8). **(H–J)** The concentration of Rgp in different groups’ serum, retina, and aqueous humor was quantitatively detected based on ELISA (*n* = 3). # Below the lower limit of detection. Statistical analysis was calculated by ANOVA. Unpaired *t*-test was used to calculate the statistical difference between the two groups. The significance (value of *p*) is defined as ^*^ *p* < 0.05, ^**^ *p* < 0.01, ^***^ *p* < 0.001, and ns *p* > 0.05.

To explore the potential impact of *P. gingivalis* OMVs on DR *in vivo*, C57 mice were injected with STZ to establish diabetes models. FFA was used to evaluate retinal pathology. Microaneurysms and fluorescein leakage were observed in 3 months diabetic mice after STZ injection (Supplementary Figure S2), suggesting the onset of DR pathology. After repeated injection of *P. gingivalis* OMVs for 8 weeks, OCT showed that hard exudates were observed in both STZ and STZ + OMVs group, as hyperreflective material in the outer retinal layers (Figure 1A). However, retinal pigment epithelium effusions were observed only in STZ + OMVs mice but not STZ alone mice (Figure 1A), indicating the increased permeability changes in the presence of *P. gingivalis* OMVs. FFA showed significant differences in microaneurysms in four groups, an increased number of microaneurysms was observed in the STZ + OMVs group than in the STZ group (Figures 1B,C). Retinal vascular digestion staining showed significant difference in pericytes count, acellular capillaries, and microvessel diameters between the different groups (Figures 1D–G). Fewer pericytes and microvessel diameters were observed in the STZ + OMVs group compared to the STZ group, and no statistical difference was observed in acellular capillaries (Figures 1D–G). The vessel diameter in OMVs group was lower than that in control group (Figure 1G). In addition, we measured the concentration of Rgp in serum, retinal and aqueous humor by ELISA. Compared with the OMVs group, higher Rgp concentrations were detected in the retinal and aqueous humor of the STZ + OMVs group, while there was no statistical difference in the serum (Figures 1H–J).

### 3.2. *Porphyromonas gingivalis* OMVs increased the expression of inflammatory cytokines, adhesion molecules, and chemokines in HRMECs *in vitro*

**Figure 2:**
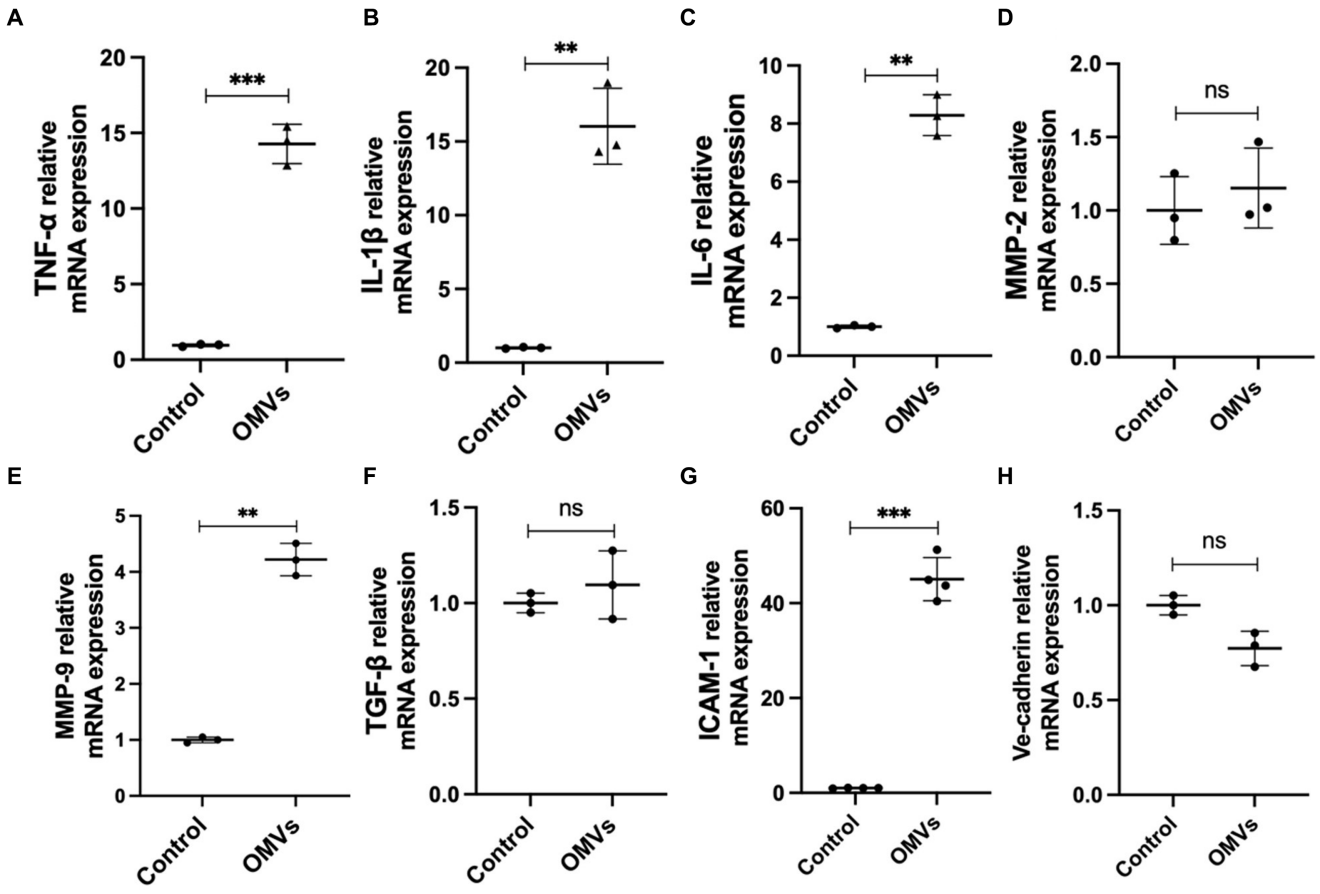
The effect of *Porphyromonas gingivalis* OMVs on HRMECs based on RT-qPCR. **(A)** TNF-α, **(B)** IL-1β, **(C)** IL-6, **(D)** MMP-2, **(E)** MMP-9, **(F)** TGF-β, **(G)** ICAM-1, **(H)** Ve-Cadherin mRNA expression are all reported. Data were shown as mean ± SD. Unpaired t-test was used to calculate the statistical difference between the two groups. When associated with the control group, significance (*p* value) was defined as ^**^ *p* < 0.01, ^***^ *p* < 0.001, and ns *p* > 0.05.

The particle size and morphology of the extracted *P. gingivalis* OMVs conformed to the distribution characteristics of *P. gingivalis* OMVs (Supplementary Figure S3). To determine the stimulating concentration of *P. gingivalis* OMVs, MTT assay was performed. The results showed that *P. gingivalis* OMVs at the final concentrations of 50 μg/mL and 100 μg/mL significantly inhibited the proliferation of HRMECs, while the 5 μg/mL and 10 μg/mL groups had no significant difference compared with the control group (Supplementary Figure S4). Therefore, 50 μg/mL *P. gingivalis* OMVs was selected as the stimulating concentration of HRMECs in subsequent experiments. In order to determine *P. gingivalis* OMV-induced cellular changes by HRMECs, gene expressions related to cellular inflammatory responses were evaluated by RT-qPCR. The mRNA levels of TNF-α, IL-1β, IL-6, MMP-9 and ICAM-1 were significantly increased in the OMVs group (Figures 2A–C,E,G), while Ve-cadherin, MMP -2 and TGF-β were not significantly different compared to the control group (Figures 2D,F,H).

### 3.3. *Porphyromonas gingivalis* OMVs increased intracellular ROS levels and induced mitochondrial dysfunction in HRMECs

**Figure 3:**
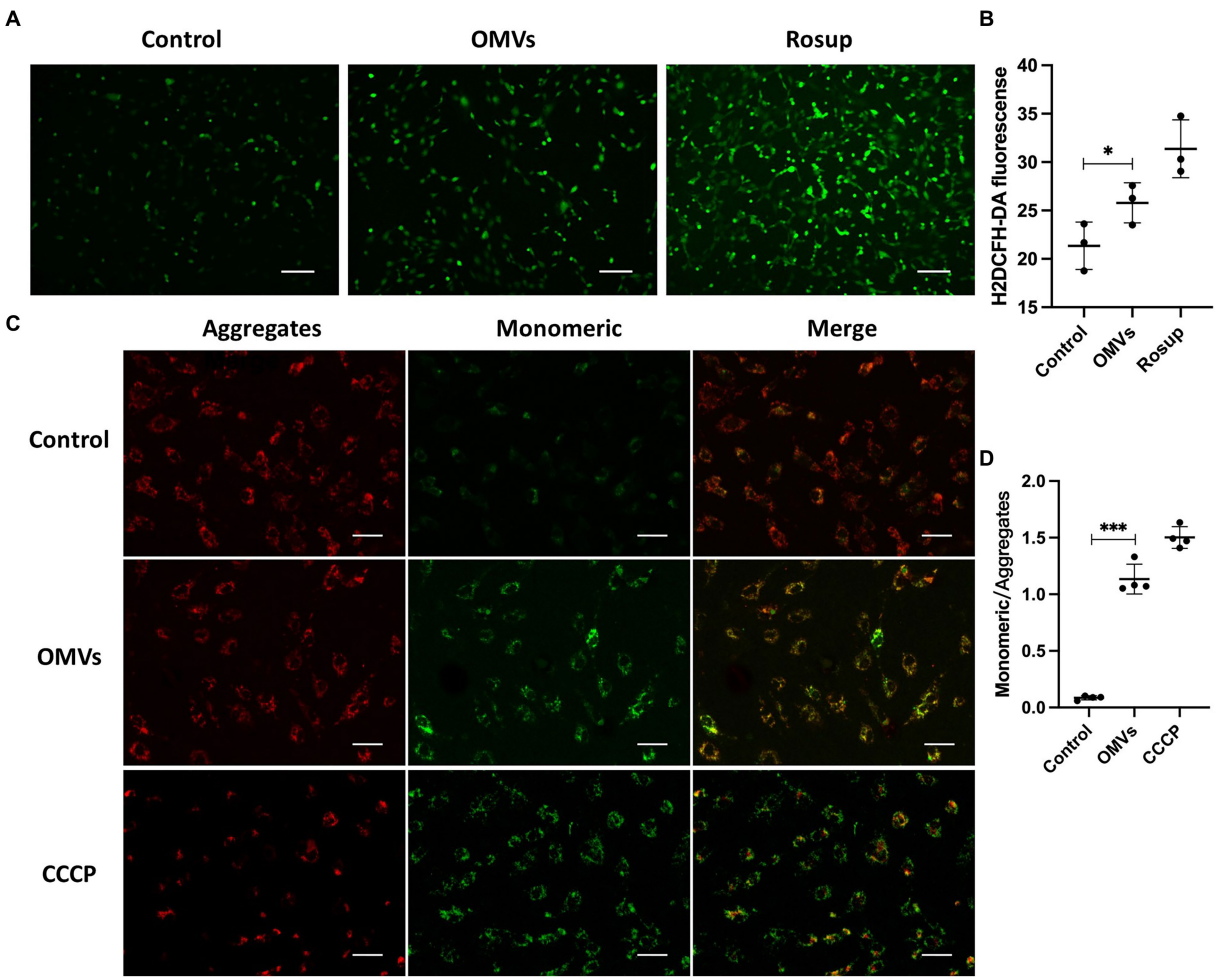
**(A)** The representative images showing the effect of the *P. gingivalis* OMVs on oxidative stress in HRMECs. Rosup was used as a positive control, and H2DCFH-DA fluorescence indicated the total ROS level in cells. **(B)** H2DCFH-DA fluorescence intensities for total ROS assay (*n* = 3). **(C)** The representative images of JC-1 staining of HRMECs after OMVs stimulation. Normal mitochondria are labeled with Aggregates fluorescence (red), and Monomeric fluorescence (green) positive areas indicate changes in mitochondrial membrane potential. Cells were treated with CCCP as a positive control. **(D)** The ratio of monomeric/aggregates fluorescence for mitochondrial membrane potential assay (*n* = 4) in each group are measured by ImageJ and shown as mean ± SD; significance was calculated by ANOVA. An unpaired *t*-test was used to calculate the statistical difference between the two groups was indicated as ^*^ *p* < 0.05 and ^***^ *p* < 0.001.

A major feature of cell death is elevated ROS levels (Villalpando-Rodriguez and Gibson, 2021). Therefore, we detected the ROS levels in HRMECs treated with *P. gingivalis* OMVs. The results showed a higher ROS level in OMVs group than in the control (Figures 3A,B). An key mechanism in DR progression is the elevation of ROS triggered by mitochondrial dysfunction, which causes cell death (Zheng et al., 2022). Therefore, we performed mitochondrial membrane potential assays to investigate mitochondrial outer membrane permeabilization. Based on the fluorescence staining, the higher fluorescence intensity of the JC-1 monomeric was observed after *P. gingivalis* OMVs stimulation (Figures 3C,D), suggesting increased mitochondrial dysfunction in HRMECs.

### 3.4. *Porphyromonas gingivalis* OMVs increased apoptosis and gene expressions of mitochondria-related cell death (MCD)

**Figure 4:**
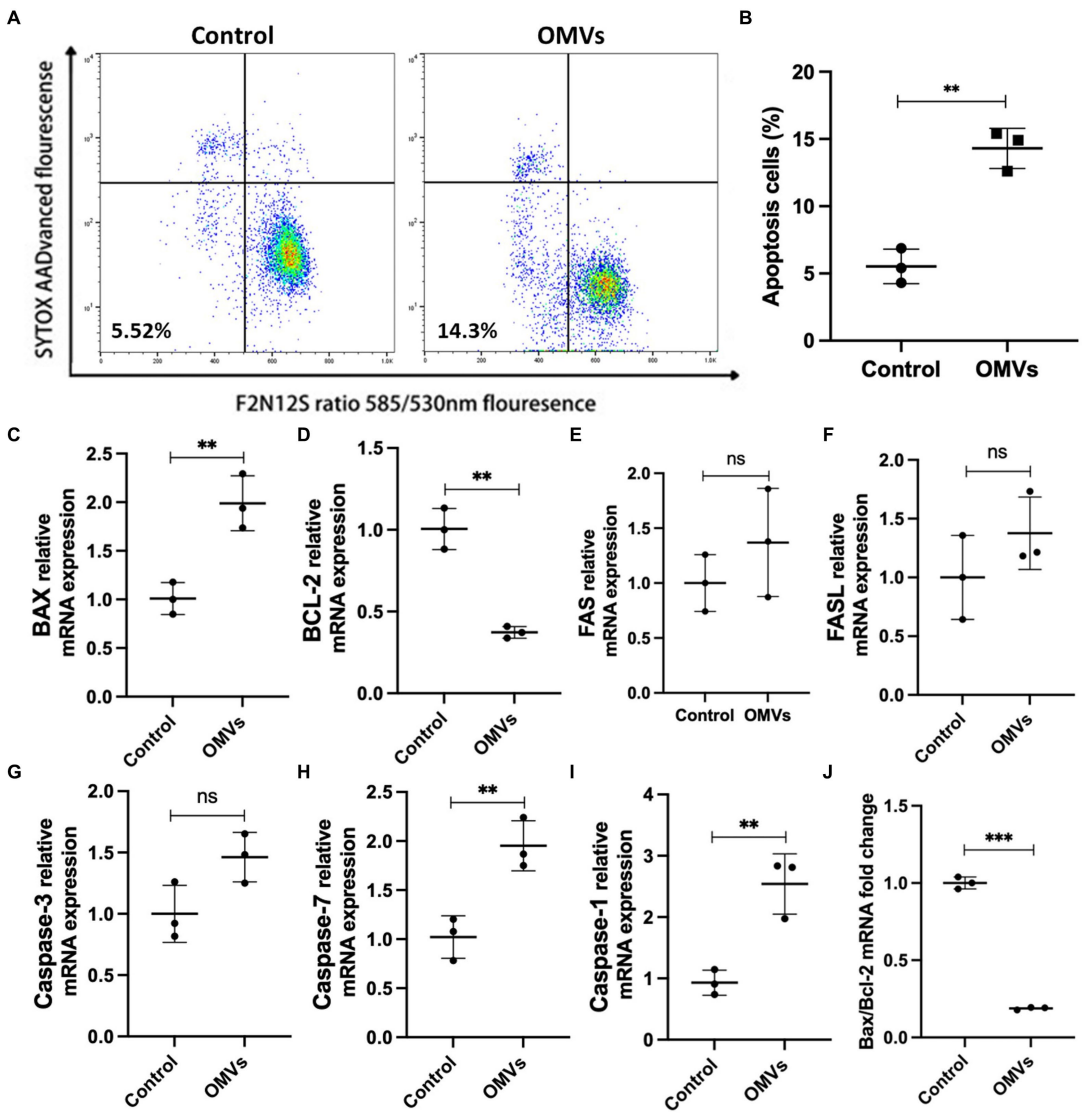
**(A)** Effect of *P. gingivalis* OMVs on HRMECs apoptosis. Flow cytometry was performed after staining with F2N12S and SYTOX AADvanced. SYTOX® AADvanced™ dead cell stain fluorescence was plotted against a derived ratio parameter from the two emission bands (585/530 nm) of F2N12S. The gate in the lower left area shows the percentage of apoptotic cells in total cells. **(B)** The percentage of apoptotic cells in HRMECs after OMVs stimulation, significance calculated by unpaired t-test is indicated as ^**^ *p* < 0.01 (*n* = 3). **(C–I)** Relative mRNA expression of factors related to cell death in HRMECs stimulated by *P. gingivalis* OMVs based on RT-qPCR. **(J)** The ratio of Bax mRNA expression to Bcl-2 mRNA expression based on RT-qPCR. Statistical analysis was calculated by unpaired *t*-test (*n* = 3). When associated with the control group, significance (value of *p*) is defined as ^**^ *p* < 0.01, ^***^ *p* < 0.001, and ns *p* > 0.05.

Next, we examined whether *P. gingivalis* OMVs cause endothelial cell death. The violet ratiometric membrane asymmetry probe/dead cell apoptosis assay was used to distinguish apoptotic cells by flow cytometry. The ratio of apoptosis cells to total cells in each group are measured. Compared with the control group, *P. gingivalis* OMVs significantly increased the apoptosis (Figures 4A,B). We further verified the mRNA expression of MCD-related genes by RT-qPCR. The mRNA expression of Bax, Caspase-1, and Caspase-7 is upregulated in OMVs group compared to the control group. An decreased in Bax/Bcl-2 gene expression rate was observed in OMVs group (Figure 4J). There is no significant difference in mRNA expression of FAS, FASL, Caspase-3 between the two groups (Figures 4C–I).

### 3.5. *Porphyromonas gingivalis* OMVs-induced inflammatory response and MCD of HRMECs Are mediated by PAR-2

**Figure 5:**
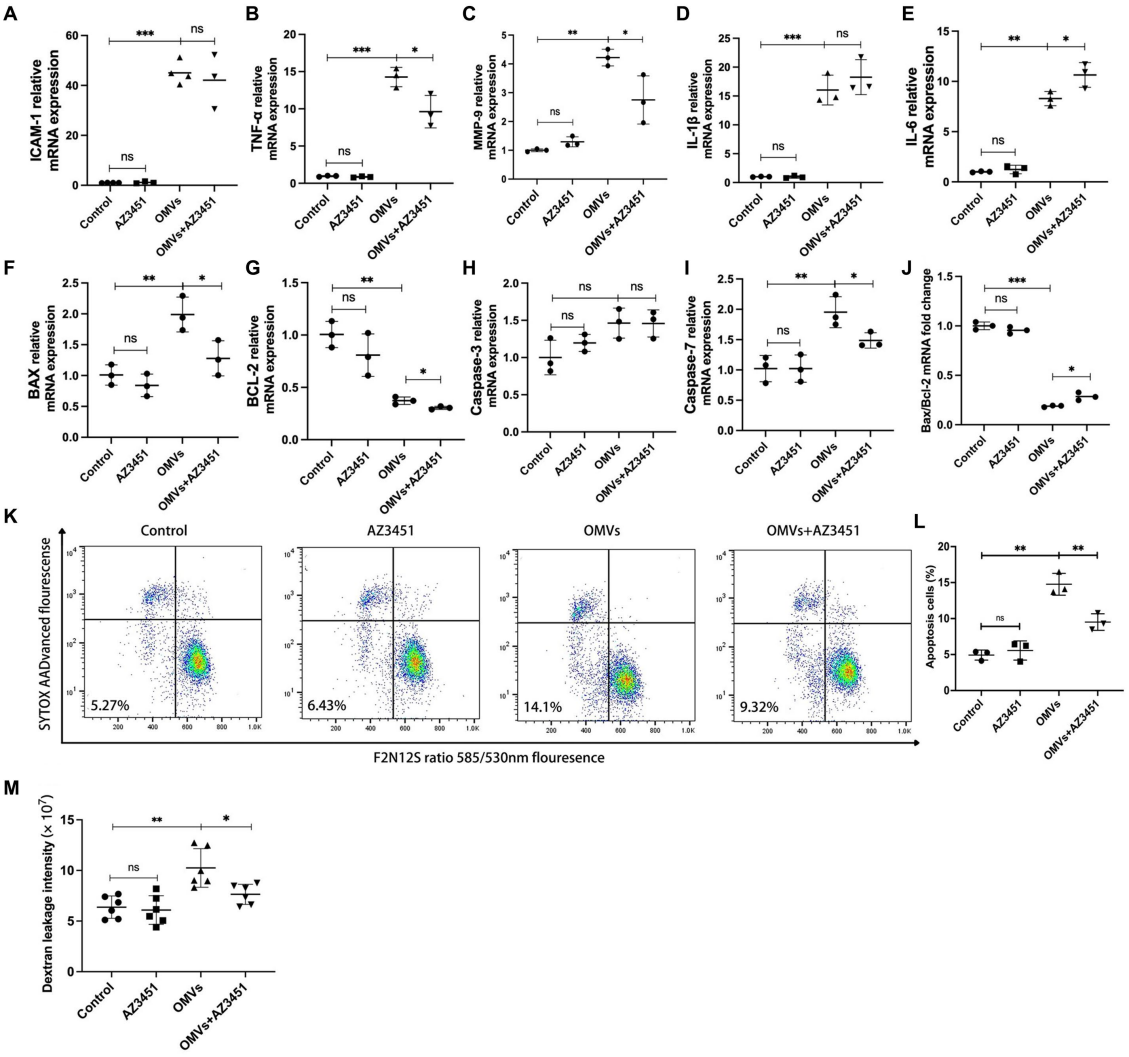
*P. gingivalis* OMVs-induced inflammatory response, MCD, and endothelial permeability are mediated by PAR-2. AZ3451, with a final concentration of 20 μM, was used as a PAR-2 antagonist to observe the effect of OMVs on HRMECs through PAR-2. Relative mRNA expression of inflammatory factors **(A–E)** and cell death-related factors **(F–I)** quantified by RT-qPCR. **(J)** The ratio of Bax mRNA expression to Bcl-2 mRNA expression based on RT-qPCR. **(K)** Flow cytometry was performed after staining with F2N12S and SYTOX AADvanced. SYTOX® AADvanced™ dead cell stain fluorescence was plotted against a derived ratio parameter from the two emission bands (585/530 nm) of F2N12S. The gate in the lower left area shows the percentage of apoptotic cells in total cells. **(L)** The percentage of apoptotic cells in HRMECs in different conditions. **(M)** After HRMECs were cultured in Transwell insert under different conditions for 24 h, 100 μg/mL Alexa Flour 488-dextran was added to the inserts and incubated for 3 h. The fluorescence intensity in the medium in the lower chamber was detected and quantified by a plate reader. Statistical analysis was calculated by ANOVA. Unpaired t-test was used to calculate the statistical difference between the two groups (*n* = 3). The significance (value of *p*) is defined as ^*^ *p* < 0.05, ^**^ *p* < 0.01, ^***^ *p* < 0.001, and ns *p* > 0.05.

In vasculature, activation of PAR-2 leads to cytokine and ROS overproduction by endothelial cells (Zhou et al., 2018; Bang et al., 2021), and previous studies indicated that activation of PAR-2 promotes inflammation and endothelial dysfunction (Zhou et al., 2018; Valencia et al., 2022). Here we investigated whether *P. gingivalis* OMVs accelerate the release of a series of inflammatory factors and promote apoptosis in HRMECs by activating PAR-2. AZ3451 was used as PAR-2 antagonist. The mRNA transcript levels of TNF-α, MMP-9, IL-1β, IL-6, and ICAM-1 were detected by RT-qPCR. Flow cytometry was used for the detection of cell apoptosis. RT-qPCR results showed significant difference of TNF-α, MMP-9, IL-1β, IL-6, and ICAM-1 mRNA expression in different groups. No changes in transcript level were observed when cells were treated with AZ3451 alone compared to the control group. The mRNA expressions of ICAM-1, TNF-α, MMP-9, IL-1β, and IL-6 were significantly upregulated when cells were treated with *P. gingivalis* OMVs compared with control (Figures 5A–E). The mRNA expression of TNF-α and MMP-9, but not IL-1β and ICAM-1, was significantly decreased in the OMVs+AZ3451-treated group as compared to OMVs group (Figures 5A–D). Interestingly, the mRNA level of IL-6 in the OMVs+AZ3451 group was higher than that in the OMVs group (Figure 5E). These results suggested that PAR-2 is involved in the process of *P. gingivalis* OMVs induced mRNA expression of TNF-α, MMP-9 and IL-6 in HRMECs. Meanwhile, the IL-1β and MMP-2 mRNA expression induced by *P. gingivalis* OMVs is independent of PAR-2. As TNF-α is upstream signal of mitochondrial dysfunction (Lv et al., 2022), and activation of MMP-9 disrupts mitochondrial homeostasis and increases retinal microvascular endothelial cell apoptosis (Kowluru and Mishra, 2017), we further determined whether PAR-2 is involved in *P. gingivalis* OMV-mediated mitochondrial dysfunction and cell apoptosis. RT-qPCR revealed that the expression of Bax, BCL-2, and Caspase-7 but not Caspase-3 was significantly different among the four groups. The mRNA levels of Bax, BCL-2, and Caspase-7 in OMVs+AZ3451 group were subsequently decreased with antagonism of PAR-2 under the *P. gingivalis* OMVs stimulation compared with OMVs group. (Figures 5F–I). Bax/Bcl-2 gene expression ratio was decreased in OMVs+AZ3451 group compared with OMVs group (Figure 5J). Based on the flow cytometry analysis, the *P. gingivalis* OMVs stimulation increase in the ratio of apoptotic cells was diminished in OMVs+AZ3451-treated group (Figures 5K,L).

### 3.6. PAR-2 mediates *Porphyromonas gingivalis* OMVs-induced elevation of endothelial permeability *in vitro*

Increased microvascular endothelial permeability is the pathological feature of early DR. Therefore, we simulated and tested the potentially disruptive effect of *P. gingivalis* OMVs on endothelial permeability *in vitro* using the Transwell system. The results showed a significant difference in endothelial permeability between the different groups. The significant increased dextran leakage was observed in OMVs group after 24 h of *P. gingivalis* OMVs stimulation compared to the control. Decreased Dextran leakage was observed in the OMVs+AZ3451-treated group compared with the OMVs group (Figure 5M).

## 4. Discussion

DR is a microvascular complication of diabetes. Histologically, disruption of the blood-retinal barrier (BRB), with early pericyte loss and endothelial damage, is followed by capillary decellularization and capillary closure, resulting in decreased blood flow and ischemia. Loss of endothelial integrity results in blood leakage, neocapillaries, and microaneurysms (Rudraraju et al., 2020). Fundus fluorescein angiography is the gold standard in DR diagnosis. We observed an increase in the number of microaneurysms in the STZ + OMVs group compared to STZ group by Fundus fluorescein angiography (Figures 1B,C). In addition, the retinal vessel staining showed a decrease in vessel diameter and pericytes (Figures 1E,G). Compared with the control group, suggesting that *P. gingivalis* OMVs have a potential destructive effect on BRB by causing endothelial dysfunction. It is worth mentioning that we observed an increase in endothelial permeability after *P. gingivalis* OMVs stimulation both *in vivo* and *in vitro*. Endothelial permeability assays in HRMECs showed that higher dextran leakage was observed in the OMVs group (Figure 5M). On the other hand, Rgp can be detected in the aqueous humor in the OMVs group *in vivo* (Figure 1I), indicating that there are permeability changes in BRB. These results confirm previous epidemiological studies on the association between periodontitis and DR (Rosenthal et al., 1988; Noma et al., 2004; Amiri et al., 2014; HR et al., 2018), suggest that periodontal infection may promote DR by releasing *P. gingivalis* OMVs. Although these results are only based on mice, it is worth considering whether *P. gingivalis* OMVs also affect the progression of DR through the same mechanism in humans? We hope that some studies based on clinical samples will further confirm this conclusion in the future.

Bacteria stimulate the host’s chronic inflammatory status by releasing OMVs into the bloodstream and enable virulence factors into deep tissues (Bomberger et al., 2009). Thus, *P. gingivalis* OMVs influence the pathogenesis and progression of periodontitis and contribute to systemic disease associated with *P. gingivalis* infection. The low-grade inflammation underlies vascular complications of DR (Forrester et al., 2020). Various molecules play a role in inducing BRB damage through multiple signaling pathways, including adhesion molecules, matrix metalloproteinases, and inflammation factors (Forrester et al., 2020). *P. gingivalis*, the major pathogen of periodontitis, is known to cause chronic low-grade systemic inflammation. In this study, *P. gingivalis* OMVs stimulation significantly increased the expression of inflammatory factor TNF-α, IL-1β, IL-6 in HRMECs (Figures 2A–C). Meanwhile, we observed significant increases in MMP-9 after *P. gingivalis* OMVs stimulation (Figure 1E ***)***. These results suggest that *P. gingivalis* OMVs may promote the progression of DR through upregulating this characteristic inflammatory factor. Previous studies have indicated that *P. gingivalis* OMVs can increase permeability by regulating intercellular junction proteins (He et al., 2020; Gong et al., 2022; Nonaka et al., 2022). Researchers found that *P. gingivalis* OMVs can change the expression of Ve-cadherin in endothelial cells at the protein level (Farrugia et al., 2020; Nonaka et al., 2022). Our results showed no differences in mRNA expression levels of Ve-cadherin (Figure 2H), which suggest that the regulation of *P. gingivalis* OMVs on Ve-cadherin may through post-transcriptional processes, such as the direct hydrolysis of proteins (Farrugia et al., 2020). Here, we explored the direct effects of *P. gingivalis* OMVs on HRMECs. In addition, the recruitment of immune cells and inflammatory response also play a key role in the progression of DR. Some studies have pointed out that OMVs can induce the recruitment and activation of immune cells (Ha et al., 2020; Lim et al., 2022). The increasing ICAM-1 expression in endothelial cells is considered the initial event of DR that promotes the adhesion of leukocytes (Hirano et al., 2010; Tang et al., 2021). Our results demonstrated that *P. gingivalis* OMVs stimulation increased the expression of ICAM-1 in HRMECs (Figure 2G). Therefore, are immune cells involved in the process of *P. gingivalis* OMVs affecting DR, and what role do they play? This is a question worth pondering in the future.

*P. gingivalis* OMVs promote endothelial dysfunction in a variety of systemic diseases (Furuta et al., 2009; Hijiya et al., 2010; Singhrao and Olsen, 2018; Seyama et al., 2020). Endothelial dysfunction caused by endothelial cell death is closely related to the occurrence and development of DR (Gui et al., 2020). The immediate manifestation is the BRB disruption due to increased endothelial permeability. Endothelial dysfunction occurs early in DR, with high ROS and mitochondrial dysfunction. ROS can up-regulate the expression of pro-inflammatory cytokines and intercellular adhesion factors in endothelial cells, which participate in the process of increased vascular permeability. A significant increase in ROS levels was observed in the OMVs group (Figures 3A,B), suggesting altered oxidative stress levels in HRMECs. Studies have pointed out that ROS is a by-product of mitochondrial metabolism, and unregulated oxidative and reductive stresses could result in severe cellular damage and cell death (Zorov et al., 2014; Rizwan et al., 2020). It is indicated that *P. gingivalis* OMVs can induce inflammation by activating cell death pathways, such as regulating BCL-2 family to cause mitochondrial dysfunction and trigger cell apoptosis (Deo et al., 2020). In our study, the expressions of Bax was upregulated after *P. gingivalis* OMVs stimulation (Figure 4C) while Bcl-2 mRNA was downregulated (Figure 4D), and Bax/Bcl-2 was decreased accordingly (Figure 4J). Meanwhile, the mitochondrial dysfunction (Figures 3C,D) and the ratio of apoptotic cells (Figures 4A,B) were increased, suggesting an elevated mitochondria-associated cell death in HRMECs. The up-regulation of Caspase-7 expression further strengthened this notion (Figure 4H). However, no statistical difference of Caspase-3 was observed between the control group and the OMVs group (Figure 4G). Some researchers pointed out that Caspase-3 and Caspase-7 play different roles in apoptosis; caspase-3 is responsible for limiting the production of ROS, and caspase-7 may contribute to the production of ROS (Brentnall et al., 2013). It suggested that the OMV-induced ROS production is mediated by Caspase-7 but not Caspase-3. The cell death signal triggered by *P. gingivalis* OMVs in HRMECs may not be mediated by FAS and FAS-L, since no changes in their expression were observed (Figures 4E,F). Notably, the expressions of Caspase-1 and IL-1β were elevated in the OMVs group (Figures 4I, 2B), indicating that pyroptosis may also be the outcome of HRMECs after *P. gingivalis* OMVs stimulation (Zeng et al., 2019). These results suggest that *P. gingivalis* OMVs can induce mitochondrial dysfunction in HRMECs and trigger cell death through multiple pathways. Overall, we proposed a possible mechanism for *P. gingivalis* OMVs to promote DR: elevated inflammatory factors led to mitochondrial dysfunction and increased ROS levels, thereby promoting the retinal microvascular endothelial cells death, resulting in endothelial dysfunction.

Protease-activated receptors (PARs) are members of the G protein-coupled receptor family. Four types of PARs have been identified in mammalian genomes. PAR-2 is the only one not activated by thrombin but activated by trypsin and tryptase (Okamura et al., 2021). Interestingly, the Gingipain derived from *P. gingivalis* has the potential to activate PAR-2 (Holzhausen et al., 2006), which is also one of the main virulence factors of *P. gingivalis* OMVs. PAR-2 is ubiquitously expressed in a variety of cells including vascular endothelial cells, and its functions involve increased vascular permeability, vasodilation, granulocyte infiltration, and cytokine release (Peach et al., 2023). There is some evidence that activation of PAR-2 is involved in the development of DR (Zhu et al., 2006). The activation of PAR-2 leads to the secretion of pro-inflammatory factors such as IL-6, TNF-α, and MMP-9, and mediates cell death (Huang et al., 2019; Carroll et al., 2021; Li et al., 2021). The up-regulated MMP-9 in mitochondria increases mitochondrial membrane permeability and promotes the entry of the pro-apoptotic protein Bax into mitochondria (Bassiouni et al., 2021; Liu et al., 2022). Our results showed that the expressions of TNF-α, MMP-9, and Caspase-7 were attenuated by PAR-2 antagonist AZ3451 (Figures 5B,C). In addition, the pro-apoptosis factor Bax/Bcl-2 was decreased (Figure 5J), and a reduced rate of apoptosis in HRMECs and improved endothelial permeability were subsequently observed after antagonized PAR-2 (Figures 5K–M). These results suggest that the increased endothelial permeability induced by *P. gingivalis* OMVs-induced inflammation and cell death is partially mediated by PAR-2, which may be the underlying mechanism by which *P. gingivalis* OMVs exacerbate DR progression. Unlike previous studies (Dekita et al., 2017; Huang et al., 2019), there is no changes in ICAM-1 were observed, and the mRNA level of IL-6 in the OMVs+AZ3451 group was higher than that in the OMVs treatment group (Figures 5A,E). This may be due to differences in antagonism methods and cell lines, and the *P. gingivalis* OMVs containing multiple products that elevate the expression of IL-6 and ICAM-1 through other receptors. The expression of IL-6 can be activated from the Toll-like 4 receptor (TLR-4) signaling pathway (Xiao et al., 2023). It is well known that *P. gingivalis* OMVs contain LPS, which can activate TLR-4. Several studies have investigated the possible connection between PAR-2 and TLR4-mediated signaling pathways. For example, concurrent activation of PAR-2 and TLR4 amplifies NF-κB activation and IL-6 production in endothelial cells, respectively (Chi et al., 2001). In contrast, TLR4-mediated inflammatory response is involved in the suppression of PAR-2 signaling (Moretti et al., 2008). Thus, signaling crosstalk between PAR-2 and TLR4 has the potential to augment or mitigate an ongoing inflammatory response when both receptors are accessible.

It is noted that our animal model was established based on studies on microcirculatory dysfunction and adopted tail vein injection of *P. gingivalis* OMVs to assess the effect in a relatively controlled time. Therefore, the results of this study are not intended to be extrapolated to fully simulate the clinical situation, where both DR and periodontitis are chronically developed conditions. Despite the limitations, this is still a viable animal model with considerable value for mechanistic studies. Future clinical investigations will be very valuable to assess the effect of *P. gingivalis* OMVs on the retinal microvascular endothelial barrier in patients with DR under chronic periodontitis conditions.

## 5. Conclusion

Our study suggested that *P. gingivalis* OMVs induced mitochondria-related cell death of HRMECs and accelerated endothelial dysfunction, thus aggravated DR, in which PAR-2 plays a potential role. To our knowledge, this study is the first research report to delineate the potential molecular mechanism of *P. gingivalis* OMVs on DR pathogenesis, which uniquely focused on the elucidation of possible impact of periodontal pathogen derivatives on DR progression. It provides a model system for expanded research in the field of microbial OMVs as a link between oral and systemic diseases.

## Data availability statement

The original contributions presented in the study are included in the article/Supplementary material, further inquiries can be directed to the corresponding authors.

## Ethic statement

The animal study was reviewed and approved by Ethics Committee of Capital Medical University.

## Author contributions

JL and XH contributed to the conception of the study and reviewed the manuscript. SH performed the research, analyzed data, and wrote the manuscript. GC, DD, and QX helped in the conduct of the study and analyzed data. SR, SS, SN, and TK assisted with the study. All authors contributed to the article and approved the submitted version.

## Funding

This research was funded by the National Natural Science Foundation of China (NSFC) grant number 82170957, and National Institutes of Health (NIH) grant number R01DE025255, DE027851, DE028715, and DE029709.

## Conflict of interest

The authors declare that the research was conducted in the absence of any commercial or financial relationships that could be construed as a potential conflict of interest.

## Publisher’s note

All claims expressed in this article are solely those of the authors and do not necessarily represent those of their affiliated organizations, or those of the publisher, the editors and the reviewers. Any product that may be evaluated in this article, or claim that may be made by its manufacturer, is not guaranteed or endorsed by the publisher.

## References

[ref1] Amiri A. A. Maboudi A. Bahar A. Farokhfar A. Daneshvar F. Khoshgoeian H. R.. (2014). Relationship between type 2 diabetic retinopathy and periodontal disease in Iranian adults. N. Am. J. Med. Sci. 6, 139–144. doi: 10.4103/1947-2714.128476, PMID: 24741553 PMC3978937

[ref2] Bang E. Kim D. H. Chung H. Y. (2021). Protease-activated receptor 2 induces ROS-mediated inflammation through Akt-mediated NF-κB and FoxO6 modulation during skin photoaging. Redox Biol. 44:102022. doi: 10.1016/j.redox.2021.102022, PMID: 34082382 PMC8182111

[ref3] Bassiouni W. Ali M. A. M. Schulz R. (2021). Multifunctional intracellular matrix metalloproteinases: implications in disease. FEBS J. 288, 7162–7182. doi: 10.1111/febs.15701, PMID: 33405316

[ref4] Bomberger J. M. MacEachran D. P. Coutermarsh B. A. Ye S. O'Toole G. A. Stanton B. A. (2009). Long-distance delivery of bacterial virulence factors by Pseudomonas aeruginosa outer membrane vesicles. PLoS Pathog. 5:e1000382. doi: 10.1371/journal.ppat.1000382, PMID: 19360133 PMC2661024

[ref5] Brentnall M. Rodriguez-Menocal L. de Guevara R. L. Cepero E. Boise L. H. (2013). Caspase-9, caspase-3 and caspase-7 have distinct roles during intrinsic apoptosis. BMC Cell Biol. 14:32. doi: 10.1186/1471-2121-14-32, PMID: 23834359 PMC3710246

[ref6] Carroll E. L. Bailo M. Reihill J. A. Crilly A. Lockhart J. C. Litherland G. J.. (2021). Trypsin-like proteases and their role in Muco-obstructive lung diseases. Int. J. Mol. Sci. 22:5817. doi: 10.3390/ijms22115817, PMID: 34072295 PMC8199346

[ref7] Chi L. Li Y. Stehno-Bittel L. Gao J. Morrison D. C. Stechschulte D. J.. (2001). Interleukin-6 production by endothelial cells via stimulation of protease-activated receptors is amplified by endotoxin and tumor necrosis factor-alpha. J. Interf. Cytokine Res. 21, 231–240. doi: 10.1089/107999001750169871, PMID: 11359654

[ref8] Dekita M. Wu Z. Ni J. Zhang X. Liu Y. Yan X.. (2017). Cathepsin S is involved in Th17 differentiation through the upregulation of IL-6 by activating PAR-2 after systemic exposure to lipopolysaccharide from Porphyromonas gingivalis. Front. Pharmacol. 8:470. doi: 10.3389/fphar.2017.00470, PMID: 28769800 PMC5511830

[ref9] Deo P. Chow S. H. Han M. L. Speir M. Huang C. Schittenhelm R. B.. (2020). Mitochondrial dysfunction caused by outer membrane vesicles from gram-negative bacteria activates intrinsic apoptosis and inflammation. Nat. Microbiol. 5, 1418–1427. doi: 10.1038/s41564-020-0773-2, PMID: 32807891

[ref10] Deshpande R. G. Khan M. Genco C. A. (1998). Invasion strategies of the oral pathogen porphyromonas gingivalis: implications for cardiovascular disease. Invasion Metastasis 18, 57–69. doi: 10.1159/000024499 10364686

[ref11] Farrugia C. Stafford G. P. Murdoch C. (2020). Porphyromonas gingivalis outer membrane vesicles increase vascular permeability. J. Dent. Res. 99, 1494–1501. doi: 10.1177/0022034520943187, PMID: 32726180 PMC7684789

[ref12] Forrester J. V. Kuffova L. Delibegovic M. (2020). The role of inflammation in diabetic retinopathy. Front. Immunol. 11:583687. doi: 10.3389/fimmu.2020.583687, PMID: 33240272 PMC7677305

[ref13] Furuta N. Takeuchi H. Amano A. (2009). Entry of Porphyromonas gingivalis outer membrane vesicles into epithelial cells causes cellular functional impairment. Infect. Immun. 77, 4761–4770. doi: 10.1128/IAI.00841-09, PMID: 19737899 PMC2772519

[ref14] Gong T. Chen Q. Mao H. Zhang Y. Ren H. Xu M.. (2022). Outer membrane vesicles of Porphyromonas gingivalis trigger NLRP3 inflammasome and induce neuroinflammation, tau phosphorylation, and memory dysfunction in mice. Front. Cell. Infect. Microbiol. 12:925435. doi: 10.3389/fcimb.2022.925435, PMID: 36017373 PMC9397999

[ref15] Gui F. You Z. Fu S. Wu H. Zhang Y. (2020). Endothelial dysfunction in diabetic retinopathy. Front Endocrinol (Lausanne) 11:591. doi: 10.3389/fendo.2020.00591, PMID: 33013692 PMC7499433

[ref16] Ha J. Y. Choi S. Y. Lee J. H. Hong S. H. Lee H. J. (2020). Delivery of Periodontopathogenic extracellular vesicles to brain monocytes and microglial IL-6 promotion by RNA cargo. Front. Mol. Biosci. 7:596366. doi: 10.3389/fmolb.2020.596366, PMID: 33330627 PMC7732644

[ref17] He Y. Shiotsu N. Uchida-Fukuhara Y. Guo J. Weng Y. Ikegame M.. (2020). Outer membrane vesicles derived from Porphyromonas gingivalis induced cell death with disruption of tight junctions in human lung epithelial cells. Arch. Oral Biol. 118:104841. doi: 10.1016/j.archoralbio.2020.104841, PMID: 32717445

[ref18] Hijiya T. Shibata Y. Hayakawa M. Abiko Y. (2010). A monoclonal antibody against fimA type II Porphyromonas gingivalis inhibits IL-8 production in human gingival fibroblasts. Hybridoma (Larchmt) 29, 201–204. doi: 10.1089/hyb.2009.0109, PMID: 20568993

[ref19] Hirano Y. Sakurai E. Matsubara A. Ogura Y. (2010). Suppression of ICAM-1 in retinal and choroidal endothelial cells by plasmid small-interfering RNAs in vivo. Invest. Ophthalmol. Vis. Sci. 51, 508–515. doi: 10.1167/iovs.09-3457, PMID: 19578010

[ref20] Hirsch I. B. Brownlee M. (2010). Beyond hemoglobin A1c--need for additional markers of risk for diabetic microvascular complications. JAMA 303, 2291–2292. doi: 10.1001/jama.2010.785, PMID: 20530784

[ref21] Holzhausen M. Spolidorio L. C. Ellen R. P. Jobin M. C. Steinhoff M. Andrade-Gordon P.. (2006). Protease-activated receptor-2 activation: a major role in the pathogenesis of Porphyromonas gingivalis infection. Am. J. Pathol. 168, 1189–1199. doi: 10.2353/ajpath.2006.050658, PMID: 16565494 PMC1606564

[ref22] Huang X. Ni B. Xi Y. Chu X. Zhang R. You H. (2019). Protease-activated receptor 2 (PAR-2) antagonist AZ3451 as a novel therapeutic agent for osteoarthritis. Aging (Albany NY) 11, 12532–12545. doi: 10.18632/aging.102586, PMID: 31841119 PMC6949101

[ref23] Kang Q. Yang C. (2020). Oxidative stress and diabetic retinopathy: molecular mechanisms, pathogenetic role and therapeutic implications. Redox Biol. 37:101799. doi: 10.1016/j.redox.2020.101799, PMID: 33248932 PMC7767789

[ref24] Kowluru R. A. Mishra M. (2017). Regulation of matrix metalloproteinase in the pathogenesis of diabetic retinopathy. Prog. Mol. Biol. Transl. Sci. 148, 67–85. doi: 10.1016/bs.pmbts.2017.02.004, PMID: 28662829

[ref25] Kumar VR S. Darisipudi M. N. Steiger S. Devarapu S. K. Tato M. Kukarni O. P.. (2016). Cathepsin S cleavage of protease-activated Receptor-2 on endothelial cells promotes microvascular diabetes complications. JASN 27, 1635–1649. doi: 10.1681/ASN.2015020208, PMID: 26567242 PMC4884104

[ref26] Li Z. X. Yang D. J. Huo Z. K. Wen P. H. Hu B. W. Wang Z. H.. (2021). Effects of chitinase-3-like protein 1 on brain death-induced hepatocyte apoptosis via PAR2-JNK-caspase-3. Biochem. Biophys. Res. Commun. 552, 150–156. doi: 10.1016/j.bbrc.2021.03.048, PMID: 33744763

[ref27] Lim Y. Kim H. Y. An S. J. Choi B. K. (2022). Activation of bone marrow-derived dendritic cells and CD4(+) T cell differentiation by outer membrane vesicles of periodontal pathogens. J. Oral Microbiol. 14:2123550. doi: 10.1080/20002297.2022.2123550, PMID: 36312320 PMC9616074

[ref28] Liu C. Liu R. Cao Z. Guo Q. Huang H. Liu L.. (2022). Identification of MMP9 as a novel biomarker to mitochondrial metabolism disorder and oxidative stress in calcific aortic valve stenosis. Oxidative Med. Cell. Longev. 2022:3858871. doi: 10.1155/2022/3858871 PMC9527114 36199424

[ref29] Lv J. Li Y. Shi S. Xu X. Wu H. Zhang B.. (2022). Skeletal muscle mitochondrial remodeling in heart failure: An update on mechanisms and therapeutic opportunities. Biomed. Pharmacother. 155:113833. doi: 10.1016/j.biopha.2022.113833, PMID: 36271583

[ref30] Moretti S. Bellocchio S. Bonifazi P. Bozza S. Zelante T. Bistoni F.. (2008). The contribution of PARs to inflammation and immunity to fungi. Mucosal Immunol. 1, 156–168. doi: 10.1038/mi.2007.13, PMID: 19079173

[ref31] HR V. Natesh S. Patil S. R. (2018). Association between diabetic retinopathy and chronic periodontitis-a cross-sectional study. Med. Sci. (Basel) 6:104. doi: 10.3390/medsci6040104 30477167 PMC6313732

[ref32] Nazir M. A. (2017). Prevalence of periodontal disease, its association with systemic diseases and prevention. Int. J. Health Sci. (Qassim) 11, 72–80. PMID: 28539867 PMC5426403

[ref33] Noma H. Sakamoto I. Mochizuki H. Tsukamoto H. Minamoto A. Funatsu H.. (2004). Relationship between periodontal disease and diabetic retinopathy. Diabetes Care 27:615. doi: 10.2337/diacare.27.2.615, PMID: 14747249

[ref34] Nonaka S. Kadowaki T. Nakanishi H. (2022). Secreted gingipains from Porphyromonas gingivalis increase permeability in human cerebral microvascular endothelial cells through intracellular degradation of tight junction proteins. Neurochem. Int. 154:105282. doi: 10.1016/j.neuint.2022.105282, PMID: 35032577

[ref35] Okamura H. Hirota K. Yoshida K. Weng Y. He Y. Shiotsu N.. (2021). Outer membrane vesicles of Porphyromonas gingivalis: novel communication tool and strategy. Jpn Dent. Sci. Rev. 57, 138–146. doi: 10.1016/j.jdsr.2021.07.003, PMID: 34484474 PMC8399048

[ref36] Peach C. J. Edgington-Mitchell L. E. Bunnett N. W. Schmidt B. L. (2023). Protease-activated receptors in health and disease. Physiol. Rev. 103, 717–785. doi: 10.1152/physrev.00044.2021, PMID: 35901239 PMC9662810

[ref37] Piano I. Novelli E. Della Santina L. Strettoi E. Cervetto L. Gargini C. (2016). Involvement of Autophagic pathway in the progression of retinal degeneration in a mouse model of diabetes. Front. Cell. Neurosci. 10:42. doi: 10.3389/fncel.2016.00042 26924963 PMC4759287

[ref38] Rizwan H. Pal S. Sabnam S. Pal A. (2020). High glucose augments ROS generation regulates mitochondrial dysfunction and apoptosis via stress signalling cascades in keratinocytes. Life Sci. 241:117148. doi: 10.1016/j.lfs.2019.117148, PMID: 31830478

[ref39] Rosenthal I. M. Abrams H. Kopczyk A. (1988). The relationship of inflammatory periodontal disease to diabetic status in insulin-dependent diabetes mellitus patients. J. Clin. Periodontol. 15, 425–429. doi: 10.1111/j.1600-051X.1988.tb01596.x, PMID: 3141483

[ref40] Rovai E. S. Holzhausen M. (2017). The role of proteinase-activated receptors 1 and 2 in the regulation of periodontal tissue metabolism and disease. J Immunol Res 2017:5193572. doi: 10.1155/2017/5193572 28503577 PMC5414592

[ref41] Rudraraju M. Narayanan S. P. Somanath P. R. (2020). Regulation of blood-retinal barrier cell-junctions in diabetic retinopathy. Pharmacol. Res. 161:105115. doi: 10.1016/j.phrs.2020.105115, PMID: 32750417 PMC7755666

[ref42] Seyama M. Yoshida K. Yoshida K. Fujiwara N. Ono K. Eguchi T.. (2020). Outer membrane vesicles of Porphyromonas gingivalis attenuate insulin sensitivity by delivering gingipains to the liver. Biochim. Biophys. Acta Mol. basis Dis. 1866:165731. doi: 10.1016/j.bbadis.2020.165731, PMID: 32088316

[ref43] Singhrao S. K. Olsen I. (2018). Are Porphyromonas gingivalis outer membrane vesicles microbullets for sporadic Alzheimer's disease manifestation? J. Alzheimers Dis. Rep. 2, 219–228. doi: 10.3233/ADR-180080, PMID: 30599043 PMC6311351

[ref44] Tang L. Zhang C. Yang Q. Xie H. Liu D. Tian H.. (2021). Melatonin maintains inner blood-retinal barrier via inhibition of p38/TXNIP/NF-κB pathway in diabetic retinopathy. J. Cell. Physiol. 236, 5848–5864. doi: 10.1002/jcp.30269, PMID: 33432588

[ref45] Teo Z. L. Tham Y. C. Yu M. Chee M. L. Rim T. H. Cheung N.. (2021). Global prevalence of diabetic retinopathy and projection of burden through 2045: systematic review and Meta-analysis. Ophthalmology 128, 1580–1591. doi: 10.1016/j.ophtha.2021.04.027, PMID: 33940045

[ref46] Valencia I. Vallejo S. Dongil P. Romero A. San Hipólito-Luengo Á. Shamoon L.. (2022). DPP4 promotes human endothelial cell senescence and dysfunction via the PAR2-COX-2-TP Axis and NLRP3 Inflammasome activation. Hypertension 79, 1361–1373. doi: 10.1161/HYPERTENSIONAHA.121.18477, PMID: 35477273

[ref47] Villalpando-Rodriguez G. E. Gibson S. B. (2021). Reactive oxygen species (ROS) regulates different types of cell death by acting as a rheostat. Oxidative Med. Cell. Longev. 2021:9912436. doi: 10.1155/2021/9912436 PMC8380163 34426760

[ref48] Xiao J. He J. He Z. Wang C. Li Y. Yan X.. (2023). Chlamydia psittaci hypothetical inclusion membrane protein CPSIT_0842 evokes a pro-inflammatory response in monocytes via TLR2/TLR4 signaling pathways. Vet. Microbiol. 280:109693. doi: 10.1016/j.vetmic.2023.109693, PMID: 36889151

[ref49] Yang Z. Tan T. E. Shao Y. Wong T. Y. Li X. (2022). Classification of diabetic retinopathy: past, present and future. Front. Endocrinol. (Lausanne) 13:1079217. doi: 10.3389/fendo.2022.1079217, PMID: 36589807 PMC9800497

[ref50] Yau J. W. Rogers S. L. Kawasaki R. Lamoureux E. L. Kowalski J. W. Bek T.. (2012). Global prevalence and major risk factors of diabetic retinopathy. Diabetes Care 35, 556–564. doi: 10.2337/dc11-1909, PMID: 22301125 PMC3322721

[ref51] Ye L. Song J. Zheng Y. Zhong M. Liu J. Zhu D.. (2022). New mechanism for mesenchymal stem cell microvesicle to restore lung permeability: intracellular S1P signaling pathway independent of S1P receptor-1. Stem Cell Res Ther 13:496. doi: 10.1186/s13287-022-03177-4, PMID: 36209115 PMC9548125

[ref52] Zeng C. Wang R. Tan H. (2019). Role of Pyroptosis in cardiovascular diseases and its therapeutic implications. Int. J. Biol. Sci. 15, 1345–1357. doi: 10.7150/ijbs.33568, PMID: 31337966 PMC6643148

[ref53] Zhang Z. Liu D. Liu S. Zhang S. Pan Y. (2020). The role of Porphyromonas gingivalis outer membrane vesicles in periodontal disease and related systemic diseases. Front. Cell. Infect. Microbiol. 10:585917. doi: 10.3389/fcimb.2020.585917 33585266 PMC7877337

[ref54] Zheng D. Liu J. Piao H. Zhu Z. Wei R. Liu K. (2022). ROS-triggered endothelial cell death mechanisms: focus on pyroptosis, parthanatos, and ferroptosis. Front. Immunol. 13:1039241. doi: 10.3389/fimmu.2022.1039241, PMID: 36389728 PMC9663996

[ref55] Zhou Q. Wang Y. W. Ni P. F. Chen Y. N. Dong H. Q. Qian Y. N. (2018). Effect of tryptase on mouse brain microvascular endothelial cells via protease-activated receptor 2. J. Neuroinflammation 15:248. doi: 10.1186/s12974-018-1287-1, PMID: 30170602 PMC6119285

[ref56] Zhu T. Sennlaub F. Beauchamp M. H.´. Fan L. Joyal J. S. Checchin D.. (2006). Proangiogenic effects of protease-activated receptor 2 are tumor necrosis factor-alpha and consecutively Tie2 dependent. Arterioscler. Thromb. Vasc. Biol. 26, 744–750. doi: 10.1161/01.ATV.0000205591.88522.d4, PMID: 16439712

[ref57] Zorov D. B. Juhaszova M. Sollott S. J. (2014). Mitochondrial reactive oxygen species (ROS) and ROS-induced ROS release. Physiol. Rev. 94, 909–950. doi: 10.1152/physrev.00026.2013, PMID: 24987008 PMC4101632

