# Supplementary material for: Porphyromonas gingivalis outer membrane vesicles exacerbate retinal microvascular endothelial cell dysfunction in diabetic retinopathy
Source: Front Microbiol. 2023 May 11;14:1167160. doi: 10.3389/fmicb.2023.1167160 (PMC10213754; doi:10.3389/fmicb.2023.1167160)
Supplement: Supplementary file 1 [file Data_Sheet_1.PDF]

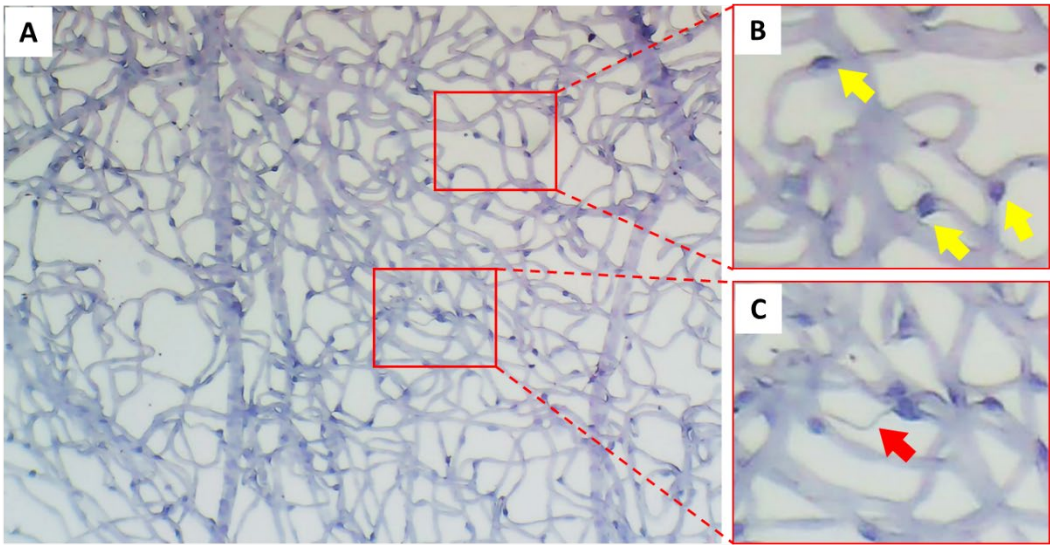

Figure S1. (**A**) The representative retinal digestion staining image of mice on the fifth month after STZ injection. (**B-C**) Partial magnification of retinal digestion staining showed the pericytes (yellow arrows) and the acellular capillary (red arrow).

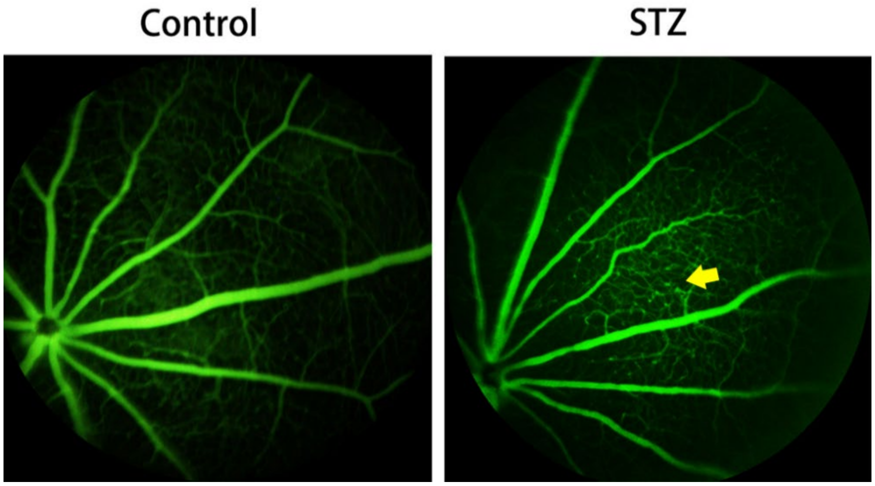

Figure S2. The representative fundus fluorescein angiography (FFA) images of mice in different groups. FFA was used to evaluate retinal pathology. Microaneurysms and fluorescein leakage were observed in the third month in diabetic mice after STZ injection (yellow arrow).

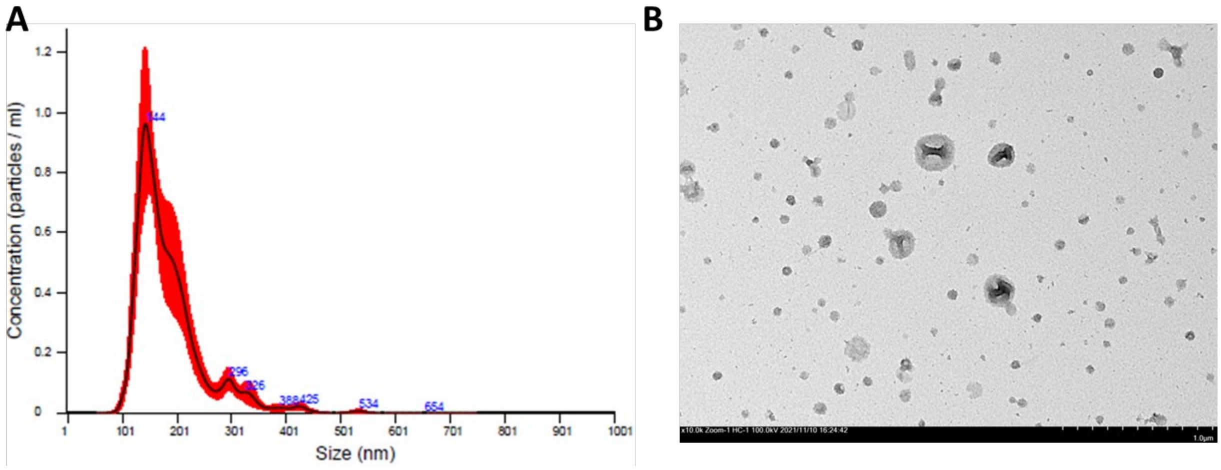

Figure S3. *P. gingivalis* OMVs particle size and morphology detection. (A) *P.gingivalis* OMVs was diluted 1000 ×, and the particle size was detected by NanoSight NS300. (B) The representative magnification transmission electron microscope (TEM) images of *P. gingivalis* OMVs. The morphology of *P. gingivalis* OMVs was observed under a 10K × TEM insight, which was consistent with the characteristics of OMVs.

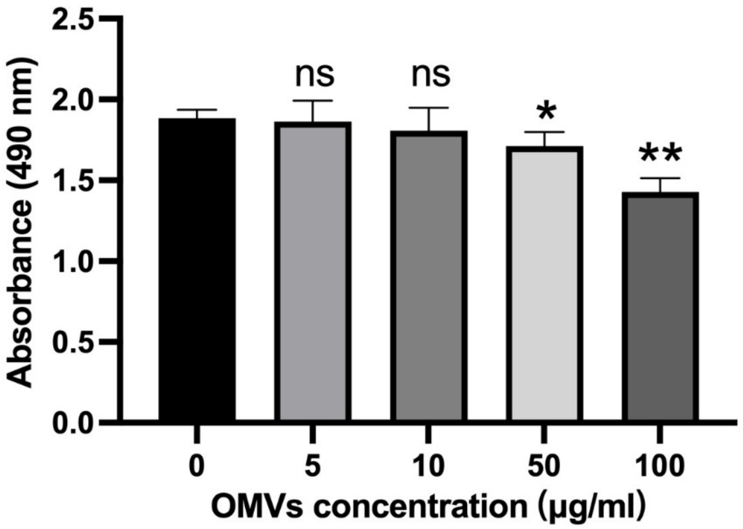

Figure S4. The effect of *P. gingivalis* OMVs on the proliferation of HRMECs was detected by MTT assay. *P. gingivalis* OMVs with final concentrations of 0 μg/ml, 5 μg/ml, 10 μg/ml, 50 μg/ml, and 100 μg/ml were added to HRMECs for 24 hours. The proliferation of HRMECs under different *P. gingivalis* OMVs concentrations stimulation were detected using the MTT assay kit according to the manufacturer's instructions. Absorbance at 490 nm was recorded by the microplate reader. ANOVA analysis showed that the proliferation of HRMECs was dose-dependent on the concentration of *P. gingivalis* OMVs. The t-test was used to analyze the statistical difference between each experimental groups and the 0 μg/ml group ($n = 3$). The significance (p-value) were marked above the column, which defined as * $p < 0.05$, ** $p < 0.01$, and ns $p > 0.05$.
